# Energy Flux Through the Magnetopause During Flux Transfer Events in Hybrid‐Vlasov 2D Simulations

**DOI:** 10.1029/2022GL100079

**Published:** 2022-10-05

**Authors:** Matti Ala‐Lahti, Tuija I. Pulkkinen, Yann Pfau‐Kempf, Maxime Grandin, Minna Palmroth

**Affiliations:** ^1^ Department of Climate and Space Sciences and Engineering University of Michigan Ann Arbor MI USA; ^2^ Department of Physics University of Helsinki Helsinki Finland; ^3^ Space and Earth Observation Centre Finnish Meteorological Institute Helsinki Finland

**Keywords:** flux transfer events, magnetopause, Vlasiator, hybrid‐Vlasov, solar wind—magnetosphere coupling, reconnection

## Abstract

Solar wind—magnetosphere coupling drives magnetospheric dynamic phenomena by enabling energy exchange between magnetospheric and solar wind plasmas. In this study, we examine two‐dimensional noon‐midnight meridional plane simulation runs of the global hybrid‐Vlasov code Vlasiator with southward interplanetary magnetic field driving. We compute the energy flux, which consists of the Poynting flux and hydrodynamic energy flux components, through the Earth's magnetopause during flux transfer events (FTEs). The results demonstrate the spatiotemporal variations of the energy flux along the magnetopause during an FTE, associating the FTE leading (trailing) edge with an energy injection into (escape from) the magnetosphere on the dayside. Furthermore, FTEs traveling along the magnetopause transport energy to the nightside magnetosphere. We identify the tail lobes as a primary entry region for solar wind energy into the magnetosphere, consistent with results from global magnetohydrodynamic simulations and observations.

## Key Points

- Energy transfer through the magnetopause experiences spatiotemporal variations under steady solar wind driving
- Energy flux within flux transfer events (FTEs) has an inward and outward component between the FTE leading and trailing edges on the dayside
- On the nightside, FTEs provide a significant energy injection to the magnetosphere

## 1 Introduction

The Earth's magnetopause is a current layer which separates the magnetospheric and shocked solar wind plasmas and their magnetic topologies, the Earth's magnetic field and interplanetary magnetic field (IMF), respectively, from each other. The interaction between the two plasmas is manifested by energy transfer through this boundary layer, which drives magnetospheric dynamics (Burton et al., 1975; Weigel et al., 2003).

Energy can exchange between the two plasmas when the magnetic topologies connect with each other. Magnetic reconnection, which rearranges the topologies and releases electromagnetic energy as kinetic and thermal energy, is a major mechanism enabling this coupling (Dungey, 1961) and most efficient during southward IMF conditions (Akasofu, 1981). Nonsteady spatially limited single reconnection sites, that is, *X*‐lines, or the occurrences of multiple quasi‐simultaneous *X*‐lines result in the formation of flux transfer events (FTEs; Fear et al., 2007; Russell & Elphic, 1978; Southwood et al., 1988), which enable energy exchange between the two plasmas by having their magnetic fields simultaneously connected to the cusp and solar wind (Paschmann et al., 1982). FTEs travel along the magnetopause transporting magnetic flux to the nightside, thereby supplying the planet's Dungey cycle (Hoilijoki et al., 2019; W. J. Sun et al., 2020) and are most frequent on the dayside and under southward solar wind conditions (Berchem & Russell, 1984). Their scale size can vary from kinetic ion‐scale structures up to the diameter of a few Earth radii (Akhavan‐Tafti et al., 2018; Eastwood et al., 2016; Fear et al., 2007).

Magnetohydrodynamic (MHD) simulations can describe energy transfer through the magnetopause at global scales (Brenner et al., 2021; Palmroth et al., 2003, 2010) and demonstrate large‐scale FTEs (Dorelli & Bhattacharjee, 2009; Fedder et al., 2002; T. R. Sun et al., 2019). In addition, the dynamics of FTEs have been previously captured in three‐dimensional (3D) hybrid‐particle in cell (PIC) simulations (Guo et al., 2021a, 2021b; Tan et al., 2011). In this study, we add to previous research by using the global hybrid‐Vlasov code Vlasiator (Palmroth et al., 2018) to construct a high‐resolution spatiotemporal assessment of the energy flux through the magnetopause during FTEs. Vlasiator includes the ion scale physics involved in magnetic reconnection capturing the reconnection rate of local plasma conditions (Hoilijoki et al., 2017).

## 2 Model and Methods

The global hybrid‐Vlasov code Vlasiator (Palmroth et al., 2018; von Alfthan et al., 2014) is a kinetic model of the Earth's magnetosphere in which 3D proton velocity distribution functions evolve according to the Vlasov equation, with electrons being a cold, massless, charge‐neutralizing fluid, and the electromagnetic fields abiding by Maxwell's equations. The closure of the system is provided by the generalized Ohm's law including the Hall term.

This study analyzes two Vlasiator simulation runs conducted in the noon‐midnight meridional Geocentric Solar Ecliptic (GSE) *XZ*‐plane, both runs being driven by radial solar wind with the flow speed of 750 km/s, the proton density of 1 cm^−3^, and the IMF of 5 nT. Simulation Run A has purely southward IMF whereas in Run B the IMF is (*B*_*X*_, *B*_*Z*_) = (cos 45°, − sin 45°) in GSE coordinates. The simulation domain in Run A (Run B) spans from −94 to 48 Earth radii (*R*_*E*_; from −48 to 64 *R*_*E*_) in the GSE *X* direction and from −56 to 56 *R*_*E*_ (from −59 to 39 *R*_*E*_) in GSE *Z*. Further information about Vlasiator and the two runs is provided in Palmroth et al. (2017, 2018) and Blanco‐Cano et al. (2018). The total simulation time of Run A (Run B) is 2,150 s (1,438 s). In this study, we analyze the time period when the magnetosphere is well‐established, that is from 1,050 to 2,150 s (from 850 to 1,438 s) for Run A (Run B).

Observations and global MHD simulations argue for the applicability of the used simulation domain and the IMF conditions: FTEs form under southward IMF conditions even if the IMF has a substantial radial component (Berchem & Russell, 1984; W. Sun et al., 2022). Moreover, during strong southward IMF conditions, such as during geomagnetic storms, the energy transfer through the magnetopause occurs predominantly in the plane parallel and antiparallel to the IMF clock angle sunward of GSE *X* > −10 *R*_*E*_ (Palmroth et al., 2003). In addition, as mentioned by Palmroth et al. (2017), a 3D full PIC simulation shows nearly two‐dimensional (2D) magnetic reconnection in the 3D system, if the reconnection guide field is zero (Pfau‐Kempf et al., 2020; Zeiler et al., 2002).

To estimate the energy transfer through the magnetopause, the magnetopause is defined by the outermost closed field line on the dayside. On the nightside, the boundary is defined by the β* parameter, where the plasma thermal pressure (*P*_th_) is supplemented by the dynamic pressure (*P*_dyn_), that is,

(1) $$\beta^{*}=\frac{P_{\text{th}}+P_{\text{dyn}}}{P_{\text{mag}}},$$

where *P*_mag_ is the magnetic pressure. The applicability of β*‐values between 0.1 and 1.5 in defining the magnetopause has been demonstrated by Brenner et al. (2021). Here, the magnetopause is given by β* = 0.1 based on visual inspection of the β* profiles. The two methods are connected at high latitudes, where we demarcate the magnetopause for distances larger than 5.7 *R*_*E*_ from the Earth, that is, beyond 1 *R*_*E*_ from the inner boundary of the simulation domain, and define the cusps as the regions where this demarcation occurs.

By following the methodology by Palmroth et al. (2003), we estimate the energy flux through the magnetopause (*K*_tot_) by computing the total energy flux **K** as

(2) $$K=S+H=\frac{E\times B}{\mu_{0}}+\frac{1}{2}\rho V^{2}V+\frac{1}{2}\sum_{j=1}^{3}p_{jj}V+\underline{p}\cdot V,$$

where **S** is the Poynting flux, **H** the hydrodynamic energy flux, **E** the electric field, **B** the magnetic field, *μ*_0_ the vacuum permeability, *ρ* the plasma mass density, *V* the plasma speed, and $\underline{p}$ the pressure tensor for the protons (Birn & Hesse, 2010). The total energy transfer rate is obtained by integrating the energy flux component normal to the boundary,

(3) $$K_{\text{tot}}=\int_{A}K\cdot dA,$$

using the sign convention such that **K** ⋅ *d* **A** is positive (negative) for energy escape from (injection into) the magnetosphere.

**Figure 1:**
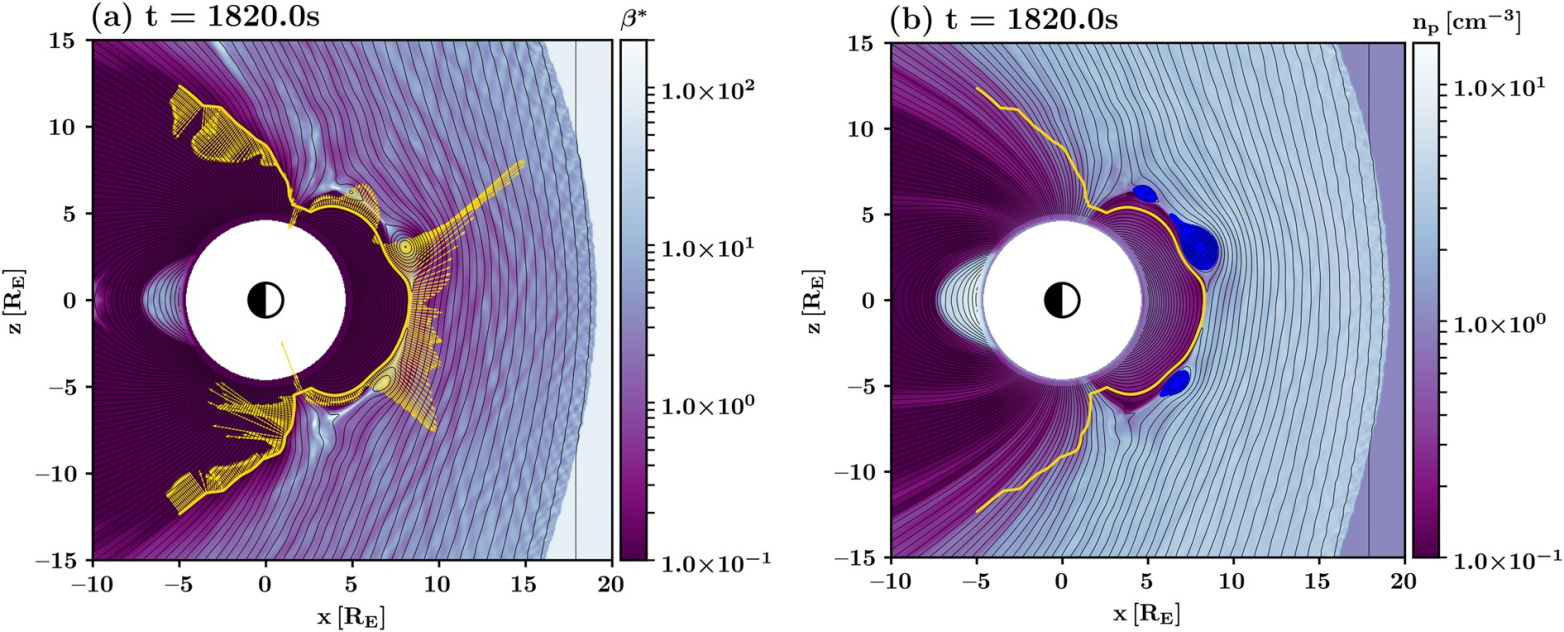
Run A at the time 1,820 s of simulation time. (a) β* in the magnetosheath and the magnetosphere (color) and the total energy flux (yellow vectors) through the magnetopause (yellow curve). The energy flux, whose absolute value varies from 6.7 ⋅ 10^−5^ to 0.26 GW, is represented by the vector length. (b) Proton number density (*n*_*p*_) in the magnetosheath and the magnetosphere. Flux transfer events given by magnetic islands in the 2D domain are marked by the blue color. The black contours of constant magnetic flux give the magnetic field lines in both panels.

Figure 1a, which shows a snapshot of Run A at simulation time *t* = 1,820.0 s, illustrates the magnetopause and the total energy flux through the boundary, with the color giving the β*. The yellow vectors exemplify the energy flux through the magnetopause (yellow curve). Small β* values distinguish the magnetosphere from the magnetosheath.

We identify FTEs in the simulation runs similar to Hoilijoki et al. (2019). In the 2D real space domain, FTEs are represented by magnetic islands and are centered at so‐called O points, which are the local maxima of the magnetic flux function Ψ(**r**, *t*)

(4) $$\Psi \left(r,\;t\right)={\left(\int_{r_{0}}^{r}B\times dl\right)}_{y},$$

where *d* **l** is the path to the examined point **r** from the reference point **r**_**0**_, which is the southern sunward corner of the simulation domain. Both the dipole magnetic field and IMF are in the simulation plane, hence no significant guide field is present in the FTEs. The FTE area (see Hoilijoki et al., 2019) becomes inaccurate at the lobes, where FTEs dissipate and reconnect with the lobe magnetic field, which is also seen in coupled kinetic—MHD simulations with embedded PIC (EPIC) calculations (Chen et al., 2017). Thus, we trace the FTE motion only from the dayside until the cusps, and limit our analysis sunward of GSE *X* > −5 *R*_*E*_. Furthermore, we focus on FTEs that persist for longer than 1 min to estimate their flux while they travel along the magnetopause. Figure 1b shows an example of FTEs and their area, with the color giving the proton number density (*n*_*p*_).

## 3 Results

**Figure 2:**
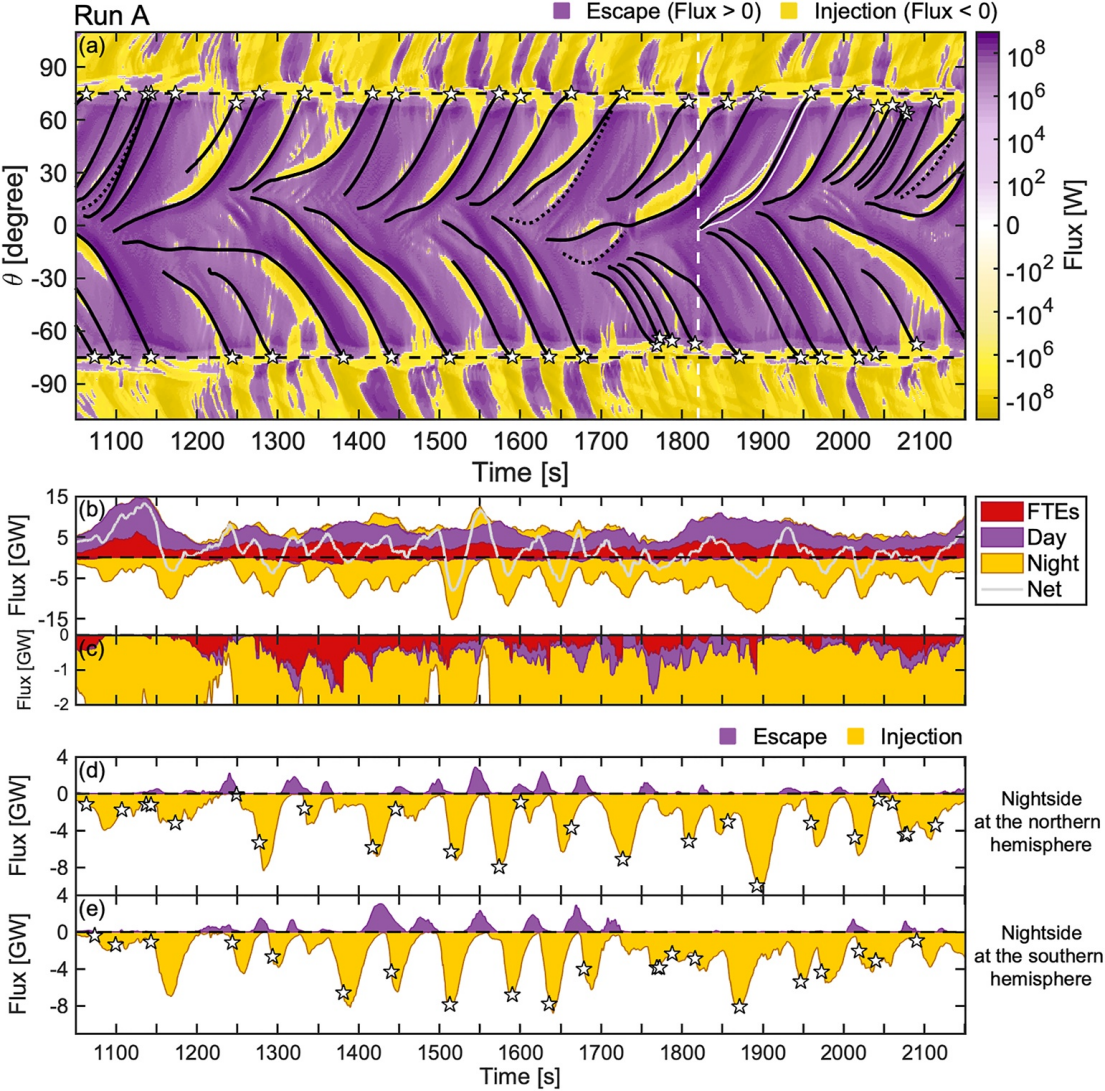
Energy flux through the magnetopause in Run A. (a) The total energy flux as a function of simulation time and polar angle (*θ*) from the subsolar nose, with the purple (yellow) indicating energy escape from (injection into) the magnetosphere. The black curves give flux transfer event (FTE) trajectories, and the black dotted curves give the trajectories of the FTEs that became merged. The cusps are indicated by the black dashed lines at *θ* = ±75°, which are the approximate center locations of the cusps at all times. The white curves exemplify the angular width of an FTE. The dashed white line indicates the time shown in Figure 1. (b) Stack plot of the integrated total energy flux computed separately for energy escape (>0) and injection (<0), which consist of FTE, dayside and nightside components. The gray curve gives the net flux including all spatial components. (c) Zoomed‐in view of panel b of the integrated inward flux. (d) Integrated energy escape (purple) and injection (yellow) through the lobe magnetopause in the northern hemisphere, and (e) in the southern hemisphere. The white stars indicate the arrival of FTEs to the cusp or their disappearance in panels (a, d, e).

**Figure 3:**
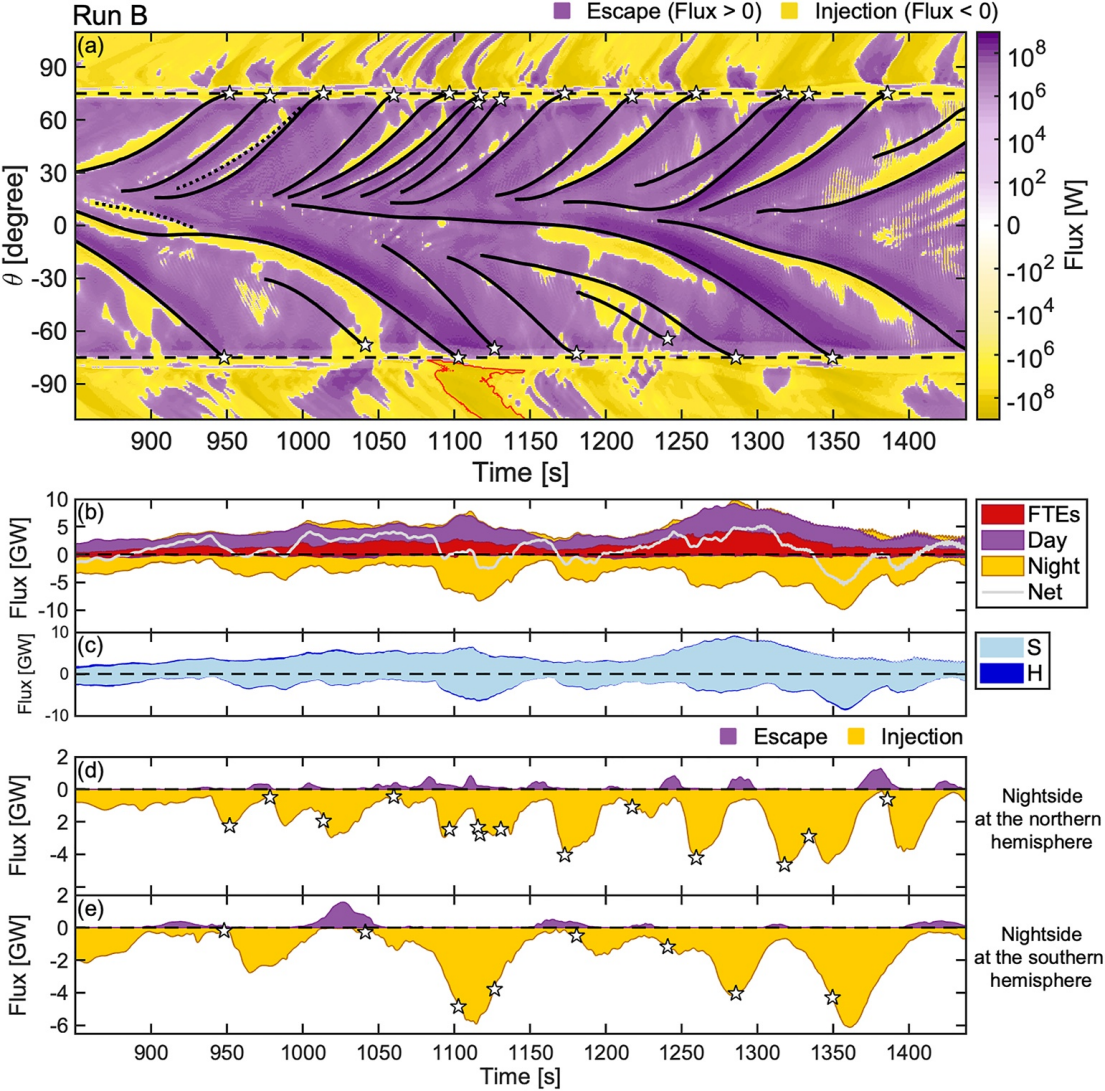
Energy flux through the magnetopause in Run B presented in a similar format to Figure 2. (a) The red contour exemplifies an enhanced energy injection on the nightside. (c) Stack plots of outward and inward fluxes, which consist of the Poynting (S) and hydrodynamic energy flux (H) components.

Figure 2a (Figure 3a) shows the total energy flux through the magnetopause (color) as a function of simulation time for a given location at the magnetopause, which is indicated by the polar angle (*θ*) relative to the subsolar nose, for Run A (Run B). The region of interest, sunward of GSE *X* > −5 *R*_*E*_, is covered by the angular interval of −115° < *θ* < 115°. The cusps are represented by the black dashed lines at *θ* = ±75°, which are the approximate center locations of the cusps at all times in both simulation runs, and used to delimit the magnetopause to the dayside and nightside. FTE trajectories are given by the black solid curves, with the white stars marking their arrival to the cusps or the time and location of their disappearance. The angular width of an FTE (characterizing its size) is shown for one sample FTE with a maximum width of 14° (2 *R*_*E*_) by the white curves enveloping the black curve in Figure 2a. The black dotted curves give the trajectories of the FTEs that became merged with another FTE.

**Figure 4:**
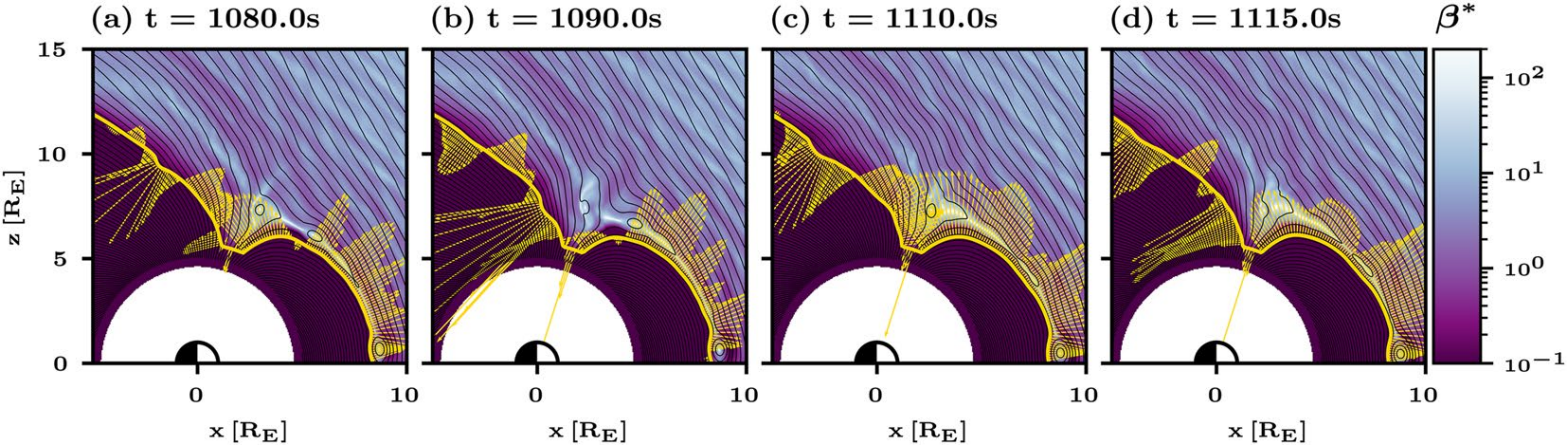
Energy flux through the magnetopause in the northern hemisphere during subsequent flux transfer events in Run B presented in a similar format to Figure 1a.

FTEs occur frequently in both simulation runs at all times as reported by Hoilijoki et al. (2019), with Run A having a more equal distribution of the FTE occurrence between the northern and southern hemispheres. In Run B with a nonzero IMF *B*_*X*_ component, FTEs are more frequent in the northern hemisphere, but the FTEs traveling south grow relatively large (see Figures 4 and 5 in Hoilijoki et al. (2019)). Most FTEs travel to the cusps and also the ones disappearing earlier reach relatively high latitudes.

Both Figures 2a and 3a show that the predominant energy flux through the magnetopause is an escape from the magnetosphere (purple, flux >0) and an injection into the magnetosphere (yellow, flux <0) between the dayside and nightside, respectively. Spatiotemporal energy injections to the magnetosphere on the dayside and the recurrent patches of energy escape on the nightside, however, differ from this trend. The injections occur at the leading edge of FTEs, whereas the escape on the nightside usually follows the arrival of an FTE to the cusp. In contrast to the leading edges, enhanced outward flows from the magnetosphere occur at the FTE trailing edges, which is demonstrated by the deeper shades of purple alongside of FTE trajectories. The inward (outward) flux at the leading (trailing) edge of an FTE traveling along the dayside magnetopause is a frequent feature of FTEs in both simulation runs (see also Figure 1a).

The predominant trends on the dayside and nightside are also distinguishable in Figure 2b and 3b, which show the stack plots of integrated total energy flux through the magnetopause as a function of simulation time computed separately for energy escape (>0) and injection (<0), which consist of three spatial components (FTE, dayside, and nightside). The FTE flux is estimated based on their angular width (see example shown by the white curves in Figure 2a). The cumulated net energy flux (gray curve) across the entire magnetopause indicates a net energy escape for both simulation runs. It is +1.9 TJ for Run A (1,100 s) and +0.6 TJ for Run B (590 s), which implies the IMF direction contributes to the energy transfer efficiency. Figure 2b and 3b also indicate that the energy escape at the FTE trailing edges surpasses the injection at the leading edges: The integrated injection within FTEs (red color) is negligible in Figures 2b and 3b compared to the escape.

The inward injected flux within FTEs, nevertheless, constitutes a significant portion of the dayside energy injection as is seen in Figure 2c, which shows the inward energy flux for Run A in a smaller scale. Most of the inward flux on the dayside not associated with FTEs occurs in the vicinity of the cusps (∼85% for 70° < |*θ*| < 80°). These findings also apply to Run B (not shown).

The absolute majority of the flux through the magnetopause is in the form of Poynting flux. Figure 3c shows the division of the flux components for Run B in a stack plot, and illustrates the vast share of the Poynting flux relative to the hydrodynamic energy flux. Approximately ∼60% of all inward hydrodynamic flux occurred at the vicinity of the cusps (65° < |*θ*| < 85°). These conclusions are similar for Run A (not shown).

We further examine the energy transfer through the magnetopause associated with FTEs by focusing on the flux on the nightside. Similarly to Figure 2b (Figure 3b), Figures 2d and 2e (Figures 3d and 3e) show the integrated total energy escape and injection in the northern and southern hemispheres (|*θ*| > 75°), respectively, for Run A (Run B). In addition, the FTE arrival times to the cusps or their disappearances are marked by white stars along the component of injection. The FTE cusp arrival predominantly precedes the peak energy surges to the magnetosphere in both hemispheres. This is also noticeable in Figure 2a (Figure 3a). In Figure 3a, a patch of such enhanced inward flux is delimited by a red contour. The surges are, furthermore, often followed by a transient energy escape at the lobes. The escapes are smaller in magnitude and coincide with the recurrent patches of outward flux in Figure 2a (Figure 3a). This is especially clear in Figure 2e at 1,350 s < *t* < 1,750 s where multiple occurrences of injections and escapes follow each other in antiphase. The surges, however, dominate the energy transfer at the lobes: The net flux is −2.6 TJ (−0.9 TJ) in the northern hemisphere and −2.5 TJ (−0.9 TJ) in the southern hemisphere in Run A (Run B).

We consider the transient outward flux that follows an FTE arrival to the cusp as the aftermath of an FTE, which is illustrated in Figure 4, which shows two FTEs traveling along the magnetopause and dissipating at the cusp in a sequence of snapshots from Run B in the northern hemisphere in a similar format to Figure 1a. In Figures 4a and 4b, an FTE reaches the cusp and is dissipated, which results in a substantial energy injection through the magnetopause at the lobe and through the cusp. The aftermath of the FTE is shown in Figure 4c, as a transient outward flux. Another energy injection to the magnetosphere occurs when the second FTE dissipates in Figure 4d. Figure 4 also suggests that the transient injections and escapes continue to travel along the magnetopause after the FTE has already disappeared. This agrees with the tilt of the patches of enhanced inward and outward fluxes on the nightside in Figures 2a and 3a.

## 4 Discussion

This study quantifies the total energy flux, which is the sum of the Poynting flux and hydrodynamic energy flux, through the magnetopause using the global hybrid‐Vlasov code Vlasiator. We analyze two simulation runs conducted in the noon‐midnight meridional GSE XZ‐plane. Run A had a purely southward IMF driving, whereas in Run B the IMF had a 45° sunward tilt (positive *B*_*X*_ together with negative *B*_*Z*_), with Run A having a greater energy transfer efficiency. In simulation runs, a net energy escape (injection) occurs on the dayside (nightside) magnetopause, which is consistent with global MHD simulation results (Brenner et al., 2021; Palmroth et al., 2003, 2010) and observations (Anekallu et al., 2013). The Poynting flux constituted an absolute majority of all flux, which may be a consequence of the 2D implementation of the Vlasiator simulation, which favors occurrence of reconnection.

We report a net escape from the magnetosphere, but the region of interest was sunward of GSE *X* > −5 *R*_*E*_ and the energy transfer was not investigated over a closed surface. The net energy escape from the magnetosphere on the dayside may be replenished by closed magnetic flux tubes that are convected from the nightside. In MHD simulations, the reconnected eroded magnetic flux on the dayside is balanced by these flux tubes, which cause a magnetic flux depletion in the near‐Earth magnetotail (Hsieh & Otto, 2014, 2015). Furthermore, plasma flows, which would convect the flux from the nightside to the dayside, have been observed (W. J. Sun et al., 2017) at the equatorial plane. Energy balance in the magnetosphere would be provided by the net energy injection on the nightside. This returning part of the Dungey cycle cannot be modeled with the present 2D implementation.

In addition to the global picture, this work assesses the contribution of FTEs to the energy transfer, resolving its spatiotemporal variations. FTEs occurred frequently in both simulation runs, with Run B having a north‐south asymmetry due to the nonzero radial IMF component. In the context of the Earth's magnetosphere, the frequency of FTE occurrence was higher in the analyzed simulation runs (Hoilijoki et al., 2019) than in MHD simulations or observations (Rijnbeek et al., 1984; T. R. Sun et al., 2019), which can result from the 2D implementation. In 2D, the IMF can pass a magnetic obstacle only by reconnecting with the obstacle's magnetic field, with the reconnection rate being constrained by the inflowing *V*_*X*_ and *B*_*Z*_. The magnetic reconnection rate in the analyzed simulation runs in this study, however, has a good correlation with an analytical model (Hoilijoki et al., 2017). In addition, the magnetic tension force accelerating FTEs can cause relatively short traveling times from the equator to the cusps in the 2D implementation. The frequency and the speeds, however, do not have an effect on the conclusions made in this study.

On the dayside, FTEs in both hemispheres traveled along the magnetopause and consisted of a leading (trailing) edge which was associated with inward (outward) flux into (from) the magnetosphere. This results from the FTE's concentric magnetic field, which defines the direction of the convectional electric field in the highly conducting plasma and regulates the direction of the Poynting flux within the FTE. Furthermore, FTE's size presumably contributes to the FTE's ability to affect the shape of the magnetopause and thus to the normal component, with larger FTEs likely resulting in larger inward and outward fluxes. Again, we note that the present setup lacks any longitudinal magnetic field component that would be present in 3D. On the nightside, FTEs reconnected with the lobe magnetic field and dissipated, which is in agreement with MHD‐EPIC and hybrid simulations (Chen et al., 2017; Omidi & Sibeck, 2007). The dissipation results in precipitating particles into the cusps (Grandin et al., 2020). Our analysis suggests that FTEs provide a significant contribution to the inward energy flux on the nightside. This is consistent with latest observations (Fear et al., 2017), and implies that FTEs are important in maintaining the magnetospheric energy balance. In addition, the injections were followed by FTE aftermaths defined as transient energy escapes from the magnetosphere, which demonstrates the local temporal variations of the flux through the magnetopause and thus the fine‐structure of the Dungey cycle.

We have demonstrated the various magnetospheric dynamics occurring during a steady solar wind driving, which in this study is manifested as spatiotemporal variations of the energy transfer through the magnetopause. Our results are in agreement with previous MHD simulations and observations. Future research focusing on how the size of FTEs and their coalescence, which alters their magnetic topologies (Akhavan‐Tafti et al., 2020; Guo et al., 2021a), affect to the energy transfer can further improve the understanding of solar wind—magnetosphere coupling.

## 5 Conclusions

In this study, we report on a high‐resolution spatiotemporal assessment of energy flux through the magnetopause during FTEs by analyzing the noon‐midnight meridional plane simulations with the global hybrid‐Vlasov code Vlasiator. We report a net energy flow out from (into) the magnetosphere on the dayside (nightside). On the dayside, FTE trailing edges contribute to the predominant outward flux. On the nightside, FTEs provide a significant contribution to the inward energy flux by reconnecting with lobe magnetic field and dissipating. In addition, we report spatiotemporal variations from the predominant flux direction, which include energy injections to the magnetosphere at FTE leading edges on the dayside and temporal energy bursts out from magnetosphere during the aftermaths of FTEs on the nightside.

## Data Availability

Vlasiator is distributed under the GPL‐2 open‐source license and uses a data structure developed at the University of Helsinki (Pfau‐Kempf et al., 2021, retrieved from https://doi.org/10.5281/zenodo.4719554). The analyzed simulation runs can be run with the aforementioned code. Alternatively, the data sets can be downloaded from the University of Helsinki servers where they are stored (Pfau‐Kempf et al., 2021). The Analysator software (Battarbee et al., 2021) was used to produce Figures 1 and 4.
